# Embracing ChatGPT for Medical Education: Exploring Its Impact on Doctors and Medical Students

**DOI:** 10.2196/52483

**Published:** 2024-04-10

**Authors:** Yijun Wu, Yue Zheng, Baijie Feng, Yuqi Yang, Kai Kang, Ailin Zhao

**Affiliations:** 1 Cancer Center West China Hospital Sichuan University Chengdu China; 2 Laboratory of Clinical Cell Therapy West China Hospital Sichuan University Chengdu China; 3 West China School of Medicine Sichuan University Chengdu China; 4 Department of Hematology West China Hospital Sichuan University Chengdu China

**Keywords:** artificial intelligence, AI, ChatGPT, medical education, doctors, medical students

## Abstract

ChatGPT (OpenAI), a cutting-edge natural language processing model, holds immense promise for revolutionizing medical education. With its remarkable performance in language-related tasks, ChatGPT offers personalized and efficient learning experiences for medical students and doctors. Through training, it enhances clinical reasoning and decision-making skills, leading to improved case analysis and diagnosis. The model facilitates simulated dialogues, intelligent tutoring, and automated question-answering, enabling the practical application of medical knowledge. However, integrating ChatGPT into medical education raises ethical and legal concerns. Safeguarding patient data and adhering to data protection regulations are critical. Transparent communication with students, physicians, and patients is essential to ensure their understanding of the technology’s purpose and implications, as well as the potential risks and benefits. Maintaining a balance between personalized learning and face-to-face interactions is crucial to avoid hindering critical thinking and communication skills. Despite challenges, ChatGPT offers transformative opportunities. Integrating it with problem-based learning, team-based learning, and case-based learning methodologies can further enhance medical education. With proper regulation and supervision, ChatGPT can contribute to a well-rounded learning environment, nurturing skilled and knowledgeable medical professionals ready to tackle health care challenges. By emphasizing ethical considerations and human-centric approaches, ChatGPT’s potential can be fully harnessed in medical education, benefiting both students and patients alike.

## Introduction

ChatGPT, whose name is derived from “generative pre-trained transformer,” is a large natural language processing model grounded in artificial intelligence (AI) technology, demonstrating remarkable performance across various language-related tasks [1]. Within the realm of medical education, ChatGPT emerges as a highly promising tool with considerable potential [2]. Through training in the ChatGPT model, medical students and doctors can enhance their clinical reasoning and decision-making capabilities, consequently leading to improved performance in case analysis and diagnosis. Moreover, ChatGPT offers personalized and efficient learning experiences for medical learners by facilitating simulated dialogues, providing intelligent tutoring, and offering automated question-answering, thereby deepening students’ comprehension of medical knowledge [3].

In the realm of transformative technologies in medical education, ChatGPT prominently distinguishes itself, standing out from other large language models by virtue of its unique architecture and comprehensive training data [4,5]. A pivotal factor setting ChatGPT apart is its monumental scale, boasting an impressive 175 billion parameters. This scale-driven proficiency contrasts starkly with smaller models that may struggle when confronted with complicated queries or tasked with producing coherent replies. With its intricate architectural foundation, ChatGPT possesses the capability to comprehend and generate human-like text across a diverse spectrum of topics, showcasing remarkable coherence and context awareness. What renders the ChatGPT truly distinctive is its specialized focus on fostering dynamic and coherent conversations, thereby excelling in maintaining context over extended interactions. This stands in stark contrast to models primarily designed for single-turn tasks. In educational contexts such as problem-based learning (PBL), team-based learning (TBL), case-based learning (CBL), and precision medical education, ChatGPT takes center stage as a focal point, primarily due to its potential to elevate dynamic learning experiences.

**Figure 1:**
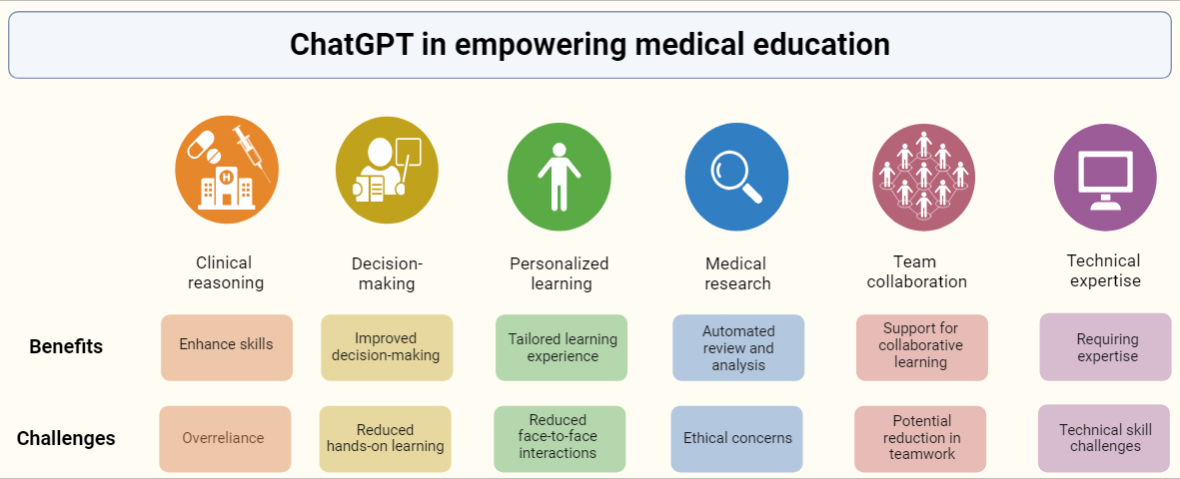
Benefits and challenges of ChatGPT in medical education.

Nevertheless, obstacles occur in the implementation of ChatGPT [6]. On the one hand, the effective training and use of the model requires a high level of technical expertise and skill. On the other hand, concerns related to data security and ethical considerations demand careful attention. To fully harness the potential of ChatGPT in medical education, these challenges must be overcome and concerted efforts should be directed toward integrating AI technology with medical education. By leveraging the capabilities of ChatGPT alongside these innovative teaching approaches, medical education can achieve new heights, fostering a generation of skilled and knowledgeable medical professionals ready to tackle the challenges of the health care field. This paper aims to illuminate both the benefits and the challenges of ChatGPT in medical education (Figure 1).

## Potential Benefits of ChatGPT in Medical Education

### Overview

In the context of medical education, ChatGPT holds immense promise for bolstering the clinical reasoning and decision-making abilities of medical students and physicians [7]. By training the ChatGPT model, medical learners can tap into its powerful natural language generation and understanding capabilities to master the methods and skills of clinical reasoning and decision-making [8,9]. These competencies are critical components of medical education and fundamental skills that medical students and physicians must possess.

### Educational Paradigms: Traditional Vs Enhanced by ChatGPT

Traditional medical education typically follows a teacher-centric approach, where the content and pace of learning are determined mainly by instructors. This often leads to passive student engagement and a lack of personalized education that caters to individual differences. However, the introduction of ChatGPT allows for more personalized and efficient medical education (Table 1). By generating learning materials based on each student’s learning status and needs, ChatGPT empowers students to take a more autonomous approach to learning and gain a customized educational experience aligned with their preferences [10,11]. For instance, students can engage in simulated dialogues with ChatGPT, discussing medical cases and diagnostic approaches. Additionally, ChatGPT can adapt based on students’ feedback and performance, providing personalized intelligent tutoring and answering questions. This personalized dialogue approach can be tailored to each student’s unique needs and interests, thereby enhancing their grasp of medical knowledge and skills.

**Table 1:** Comparison between traditional medical education and medical education with ChatGPT.

Aspect	Traditional medical education	Medical education with ChatGPT
Clinical reasoning	Instructor-led lectures and traditional case discussions	Enhanced clinical reasoning, personalized dialogues, and simulated case analyses
Decision-making	Limited case exposure	Diverse cases and diagnostic approaches
Personalized learning	One-size-fits-all learning materials and standardized assessments	Tailored learning materials, intelligent tutoring, and automated question-answering based on individual progress
Interaction with educators	Limited face-to-face interactions	Continuous personalized feedback
Medical research support	Manual review and analysis	Automated literature review, study design proposals, and statistical analysis
Team collaboration	Emphasizes group discussions and teamwork	Balancing personalized learning and team-based activities
Technical expertise and challenges	Less reliance on technology	Skill in using ChatGPT
Ethical considerations	Data privacy and consent	Addressing ethical implications

### ChatGPT Intelligent Tutoring in PBL Integration

The integration of ChatGPT holds promising implications for PBL in medical education. ChatGPT’s capacity to offer personalized guidance and stimulate critical thinking aligns seamlessly with the core principles of PBL [12]. In this context, ChatGPT functions as an intelligent tutor, adept at steering students through intricate problems by furnishing pertinent information, detailed explanations, and insightful suggestions. The model’s ability to dynamically adjust responses to student queries contributes to creating a vibrant and responsive learning environment. Students can leverage ChatGPT to brainstorm potential solutions, collect relevant research, or validate hypotheses during the problem-solving process [13]. Furthermore, the model can generate patient cases or clinical scenarios based on real-world data, enabling students to apply their knowledge to practical situations. It is essential to design PBL activities that seamlessly incorporate both the advantages offered by ChatGPT and the indispensable experience derived from clinical practice. By maintaining a focus on group discussions and collaborative problem-solving based on actual patient cases, educators ensure that students reap the benefits of ChatGPT’s enhancements while retaining the essential skills cultivated through hands-on clinical interactions and in-depth case analyses. As technology continues to advance, it remains imperative to uphold patient-based learning as the cornerstone of medical education. Recognizing that, at its current stage, ChatGPT cannot entirely replace the critical skills honed through genuine patient interactions and the nuanced analysis of complex cases is vital for preserving the integrity and effectiveness of medical education.

### Synergizing ChatGPT With Other Collaborative Teaching Methods

ChatGPT’s application in medical education should be complemented by other teaching methods, such as CBL, TBL, and small-group sessions. The model’s ability to generate diverse perspectives and solutions enhances the overall TBL experience [14]. In CBL scenarios, ChatGPT can function as a case facilitator, generating realistic scenarios, asking probing questions, and providing nuanced feedback. It can simulate authentic patient interactions or complex business dilemmas, allowing learners to apply theoretical knowledge to practical situations. The model’s adaptability ensures that the cases presented are tailored to the evolving needs and understanding of the learners. Within the TBL framework, ChatGPT can facilitate collaboration among team members by offering real-time assistance and promoting knowledge sharing. It can contribute to group discussions, help clarify concepts, and prompt critical thinking among team members. ChatGPT can also facilitate preclass preparation by providing students with foundational knowledge and resources related to the upcoming TBL session. By integrating ChatGPT with these methods, medical educators can create a well-rounded learning experience that maximizes the benefits of both individualized learning and TBL. To enhance team collaboration abilities, medical institutions should prioritize the development of medical students through interprofessional education, where students from different health care disciplines collaborate. Encouraging student-led initiatives and group projects also fosters collaboration, leadership, and effective communication among future medical professionals. This multifaceted approach ensures a well-rounded learning experience, maximizing the benefits of both individualized and collaborative learning while preparing students for the complex challenges of the health care field.

### ChatGPT in Precision Medical Education

In the evolving landscape of medical education, the concept of precision medical education has gained prominence [15]. This approach aligns with current trends, notably competency-based medical education (CBME) and pedagogical approaches such as PBL, CBL, and TBL [16]. Precision medical education emphasizes tailoring learning experiences to individual student needs, aligning with the principles of personalized and adaptive learning championed by ChatGPT. CBME focuses on learners progressing at their own pace, demonstrating proficiency in specific competencies. ChatGPT's intelligent tutoring and adaptability make it a valuable tool in supporting this competency-based model. By providing personalized guidance, generating relevant content, and fostering critical thinking, ChatGPT contributes to a more precise and effective medical education tailored to each learner’s requirements [17]. Furthermore, the integration of ChatGPT with collaborative teaching methods enhances the multifaceted nature of precision medical education. In scenarios like CBL and TBL, ChatGPT assists learners in navigating complex medical cases, fostering collaborative problem-solving skills essential for modern health care practice. This approach ensures that students not only acquire essential competencies but also develop the ability to collaborate across health care disciplines, aligning with the interprofessional education framework.

As medical education continues to advance, the incorporation of precision medical education, supported by technologies like ChatGPT, becomes imperative. This tailored approach ensures that medical professionals are equipped with the diverse skills needed to address the complexities of contemporary health care, providing a comprehensive and forward-thinking educational experience.

### Empowering Medical Research With ChatGPT

ChatGPT proves to be a valuable asset in medical research [18]. The intricate relationship between medical research and education, as aligned with the standards and roles outlined by the World Federation for Medical Education (WFME) [19] and Canadian Medical Education Direction System (CanMEDS) [20], not only provides a profound and practical foundation for medical education but also aligns with the comprehensive development requirements for medical professionals. This close connection ensures that medical education remains consistent with the latest advancements in medical science, fostering the cultivation of well-rounded medical practitioners. Medical research relies heavily on extensive literature to support its content and conclusions. However, reading and analyzing vast amounts of literature can be time-consuming and labor-intensive. ChatGPT streamlines research by automating literature review and analysis. Additionally, ChatGPT aids medical researchers in study design and data analysis [21]. By expediting data processing, extracting data features and patterns, generating research design proposals, and offering statistical analysis methods and data visualization tools, ChatGPT facilitates improved experiment design and data analysis.

## Challenges of ChatGPT in Medical Education

### Overview

While ChatGPT offers substantial benefits to medical education, it faces a spectrum of challenges [22]. The rapid pace of knowledge evolution within the medical field presents a significant hurdle. New research and clinical guidelines continually emerge, demanding constant updates to ChatGPT to ensure that students are provided with the most current and accurate medical information. This necessitates not only the ability to keep up with knowledge updates but also to ensure their accuracy and credibility.

### Potential Devaluation of Collaboration

A notable concern emerges regarding the potential devaluation of the collaborative aspect of learning in medical education, particularly in traditional methodologies such as PBL, CBL, and TBL. Collaboration and teamwork are pivotal in these approaches [23], and ChatGPT may inadvertently diminish the importance of human-to-human interaction. Maintaining a balance between technology and interpersonal relationships is vital for effective learning. While ChatGPT enhances PBL through personalized guidance, educators must underscore the enduring importance of patient-based learning and teamwork. Despite its simulation capabilities and theoretical insights, ChatGPT cannot replace practical experiences gained through real-world interactions, especially in medical education. Acknowledging the model’s limitations is crucial to prevent an overreliance on simulated learning. Embedding ChatGPT seamlessly into existing curricula presents a challenge, requiring educators to invest time in designing and integrating AI-driven components aligned with overall learning goals.

### Overreliance

Importantly, overreliance on technology may hinder critical thinking and hands-on learning, potentially lowering the quality of education. ChatGPT’s answers can vary or even contradict themselves with each query, further impacting student learning [24]. Learning through ChatGPT might inadvertently reduce face-to-face interactions with educators and peers, impacting effective communication skills in clinical practice. ChatGPT may occasionally disseminate inaccurate medical information, making the prompt recognition and correction of such errors critical [25,26]. The establishment of supervision and feedback mechanisms to enhance ChatGPT’s accuracy is imperative.

### Challenge of Personalized Learning

The challenge of personalized learning is a crucial consideration. Every student has distinct needs and academic levels, requiring ChatGPT to offer tailored education that aligns with individual requirements and progress. Achieving this may necessitate the development of more sophisticated algorithms and technologies. Cultural diversity and inclusivity should also be addressed. Medical education needs to accommodate students from different cultural backgrounds. ChatGPT should be capable of delivering information and using teaching methods that ensure effective comprehension and benefits for all students.

### Ethical Considerations

The ethical and privacy dimensions of using ChatGPT in medical education are paramount [27,28]. Handling patient data in an educational context and safeguarding patient privacy are complex and vital concerns. This entails strict adherence to regulatory and ethical guidelines. Identifying and rectifying errors is another noteworthy challenge.

### Technological Accessibility

Technological accessibility poses a challenge. The effective use of ChatGPT depends on network connectivity and device availability, which can be problematic in various regions and among specific student populations [29]. Strategies must be devised to use ChatGPT in diverse technological environments.

## Future Directions of ChatGPT in Medical Education

### Overview

To mitigate these issues, appropriate regulation and supervision are essential. Students should receive training in interpersonal interactions to engage effectively with patients and efforts should be made to provide equal access to technology and learning resources, promoting fair and inclusive medical education. Moving forward, research in this field should explore various promising avenues to enhance our comprehension and application of ChatGPT.

### Strategies to Tackle Present Challenges

To specifically address the challenges of ChatGPT on PBL, TBL, and CBL, measures should be taken to mitigate potential drawbacks on collective capabilities. Introducing targeted interventions, such as incorporating collaborative exercises and feedback mechanisms, can help balance individual contributions within a team setting. Emphasizing the importance of teamwork in medical education [30], alongside the integration of ChatGPT, can foster a collaborative learning environment.

There is a pressing need to investigate methods that can augment ChatGPT’s capacity to deliver contextually relevant and up-to-date medical information. This involves developing mechanisms for real-time knowledge updates and refining the curation of medical data. Besides, it is crucial to address the ethical and privacy challenges associated with ChatGPT [31]. Future research can focus on devising robust protocols and AI-driven solutions to protect patient data while seamlessly integrating ChatGPT into medical education. Furthermore, exploring innovative approaches for personalizing medical education with ChatGPT presents an exciting opportunity. Research can delve into adaptive learning algorithms and inventive teaching strategies tailored to individual student needs and learning styles. Additionally, there is a need for research on improving ChatGPT’s error identification and correction mechanisms, ensuring the highest level of accuracy and reliability in medical content. Finally, we should examine ways to enhance ChatGPT’s cultural sensitivity and inclusivity in medical education and acknowledge the diversity of student backgrounds and learning requirements. This holistic approach ensures that ChatGPT not only provides accurate medical information but also aligns with the broader goals of medical education in promoting collaboration, ethical considerations, and cultural competence.

### Conclusions

In conclusion, ChatGPT enhances medical education by improving clinical reasoning, personalizing learning, promoting precision medical education, and supporting medical research. However, a balanced and responsible integration requires a focus on ethics and human-centered approaches. Medical educators can achieve this balance by customizing learning paths, blending personalization with group activities, assigning team projects, guiding ChatGPT use, and emphasizing ethics and critical thinking training. These steps create a holistic learning environment that prepares students to excel as independent thinkers and team players in health care, optimizing ChatGPT’s role in medical education while maintaining its integrity.

## Abbreviations

AI: artificial intelligence
CanMEDS: Canadian Medical Education Direction System
CBL: case-based learning
CBME: competency-based medical education
PBL: problem-based learning
TBL: team-based learning
WFME: World Federation for Medical Education
